# Comparison of short-term recovery in children with obstructive sleep apnea undergoing tonsillotomy vs. tonsillectomy

**DOI:** 10.3389/fped.2022.969973

**Published:** 2022-10-25

**Authors:** Yanwei Dang, Yujie Li, Junbo Zhang, Wei Huang, Yuke Dong, Xiao Shen, Wei Han, Tao Li

**Affiliations:** ^1^ Department of Otolaryngology, Head and Neck Surgery, Zhengzhou Central Hospital Affiliated to Zhengzhou University, Zhengzhou, China; ^2^ Department of Otolaryngology, Head and Neck Surgery, Peking University First Hospital, Beijing, China

**Keywords:** child, coblation, tonsillotomy, tonsillectomy, pain

## Abstract

**Objectives:**

To compare the pain levels, degrees of pharyngeal swelling, and weight loss after tonsillectomy vs. tonsillotomy in children clinically diagnosed with obstructive sleep apnea (OSA) over the first seven postoperative days, and to determine which procedure was associated with better recovery in the early postoperative period.

**Methods:**

Between April 2021 and December 2021, 121 children with OSA (80 males and 41 females), ranging from 3 to 12 years of age with an average age of 6.7 years, were prospectively enrolled in this study conducted at Zhengzhou Central Hospital Affiliated to Zhengzhou University. The patients were randomly divided into two groups: a tonsillotomy group with 63 cases (40 males and 23 females) and a tonsillectomy group with 58 cases (40 males and 18 females). The patients' pain levels [as indicated by Parents' Postoperative Pain Measure (PPPM) scores] and degrees of pharyngeal swelling were recorded for seven days postoperatively, and the patients' body weights were recorded on postoperative day seven.

**Results:**

In the tonsillotomy group, the PPPM scores were the highest on the day of surgery and on the first postoperative day; the patients' pain levels gradually decreased.The PPPM scores in the tonsillectomy group were higher than those in the tonsillotomy group from the day of surgery to the seventh postoperative day (*p* < 0.05). The degree of pharyngeal swelling was lower in the tonsillotomy group than in the tonsillectomy group. Weight loss was lower in the tonsillotomy group than in tonsillectomy group on the 7th day after surgery (*p* < 0.05). On the fifth, sixth, and seventh postoperative days, compared with preschool children, school-age children who had undergone tonsillotomy experienced more pain relief than those who had undergone tonsillectomy (*p* < 0.05).

**Conclusion:**

Children with OSA experienced less pain, less pharyngeal swelling, and less weight loss with tonsillotomy than with tonsillectomy. On the fifth, sixth, and seventh postoperative days, compared with preschool children, tonsillotomy in school-age children is more advantageous in school-age children.

## Introduction

Obstructive sleep apnea (OSA) refers to a series of pathophysiological changes caused by frequent partial or total upper airway obstruction during sleep. The common cause is tonsil and/or adenoid hypertrophy (1, 2). Tonsillectomy and/or adenoidectomy are the treatment methods. After tonsillectomy, patients suffer from long-lasting severe pain. With the development of minimally invasive technologies, such as plasma and ultrasonic scalpels, in Western countries, tonsillotomy has gradually replaced tonsillectomy for the treatment of OSA in children (3, 4). In this study, continuous observations and comparisons of pharyngeal pain were performed to investigate the changes in pain levels after the two surgical procedures.

## Patients and methods

### Study subjects

This prospective study involved patients undergoing tonsil and adenoid surgery for OSA at Zhengzhou Central Hospital Affiliated to Zhengzhou University from April 2021 to December 2021. The inclusion criteria were (1) the diagnostic criteria of the Chinese Guideline for the Diagnosis and Treatment of Childhood Obstructive Sleep Apnea (2020) (5), (2) Before deciding on surgery, 90.3% of patients received regular conservative treatment. Complete preoperative polysomnography, OSA diagnosis, and snoring with mouth breathing during sleep, and (3) in line with the indications for tonsillectomy and adenoidectomy, tonsils of two degrees or more, and electronic nasopharyngoscopy or computed tomography indicating pathological adenoid hyperplasia(the pathological hypertrophy of adenoid is defined as 2/3 or more of the nostril after obstruction by adenoid tissue under nasal endoscopy or CT shows that A/N is greater than or equal to 0.7). The exclusion criteria were (1) recurrent tonsillitis (more than seven times over the previous year or more than five times per year for two consecutive years or more than three times per year for three consecutive years), (2) craniofacial deformity, (3) bleeding diathesis(Hemophilia, VK deficiency, immune thrombocytopenia, etc), (4) immunodeficiency, and (5) acute tonsil infection.

According to the inclusion and exclusion criteria, 121 patients (80 males and 41 females) were included in the study. Sample size calculation for the study: n1 = n2 = 4[(t a/2 + t ß)2 S2]/*δ*2. The patients' ages ranged from 3 to 12 years, and their average age was 6.7 years.

The patients were randomly divided into a tonsillotomy group and a tonsillectomy group. The tonsillotomy group included 63 cases (40 males and 23 females) with an average age of 6.6 years. Among them, 30 children were of preschool age (3–6 years old), and 33 were of school age (7–12 years old). The tonsillectomy group consisted of 58 cases (40 males and 18 females) with an average age of 6.9 years. Among them, 26 children were of preschool age, and 32 were of school age. There were no significant differences in baseline data, such as age, gender, and body mass index, between the two groups (all *p* > 0.05; Table 1). Informed consent was obtained from all patients' legal guardians. This study was approved by the hospital's ethics committee, The detailed number of the ethical application process: 202080.

**Table 1:** Basic demographic information on the two patient groups.

Variable	Tonsillotomy group (*n* = 63)	Tonsillectomy group (*n* = 58)	*χ*^2^/t	*P*
Sex, *n*			0.404 (χ^2^)	0.525
Male	40	40		
Female	23	18		
Age (years), mean ± SD	6.57 ± 2.18	6.95 ± 2.59	−0.869 (t)	0.387
Body mass index, mean ± SD	0.65 ± 0.17	0.64 ± 0.18	0.334 (t)	0.739

### Surgical procedures

Under general anesthesia and endotracheal intubation, all patients were placed in the supine position. An open mouth gag was used to fully expose the oropharynx. A low-temperature plasma system was used to assist in the operation. The nasopharynx was exposed through the oropharynx, and the adenoids were removed under the guidance of a 70° endoscope. In the tonsillotomy group, low-temperature plasma was used to ablate the tonsils, layer by layer, from their upper pole surface to the tonsillar fossa's thin layer of tonsil tissue. In the tonsillectomy group, low-temperature plasma was used to completely remove the tonsils along the tonsillar capsule.

### Pain

The Parents' Postoperative Pain Measure (PPPM) is mainly used to assess pain-related behavioral changes in children aged 1–12 years (6) and includes 15 items:

1. Is the child more likely to complain than usual?
2. Is the child crying more than usual?
3. Is the child playing less than usual?
4. Does the child not enjoy doing what they usually do?
5. Is the child more anxious than usual?
6. Is the child quieter than usual?
7. Is the child less energetic than usual?
8. Is the child eating less than usual?
9. Does the child cover the painful area?
10. Does the child refuse to eat?
11. Is the child afraid to touch the painful area?
12. Is the child groaning more than usual?
13. Does the child prefer to be close to you?
14. Does the child take drugs that are usually refused?
15. Is the child's face redder than usual?

Each item is answered with a “yes” or a “no.” A “yes” corresponds to 1 point, while a “no” corresponds to zero points. The points are then added to give a total score. The total score ranges from 0 to 15. A score of ≥6 indicates severe pain. The patients’ parents completed the PPPM daily from the day of surgery (Day 0) to the seventh postoperative day (Day 7).

### Degree of pharyngeal swelling

Pharyngeal swelling from Day 0 to Day 7 was scored as follows: 0: no swelling of the palatopharyngeal arch, palatoglossal arch, or uvula; 1: edema limited to the area around the tonsillar fossa; 2: edema spread around the tonsillar fossa and soft palate, but no swelling of the uvula; 3: edema spread around the tonsillar fossa, soft palate, and uvula, but relatively normal shape of the uvula; 4: edema spread around the tonsillar fossa, soft palate, and uvula, and uvula swollen to a spherical shape.

### Weight

Each participant's body weight was recorded before surgery and 7 days after surgery.

### Statistical analysis

IBM SPSS Statistics 22.0 software was used for the statistical analysis. The Shapiro–Wilk test was used to assess data normality. Age, BMI, PPPM scores of patients in the two groups on the day of surgery, the first, second day after surgery, the degree of pharyngeal swelling on the day of surgery, the first, second, third day after surgery were in line with normal distribution, the normal distribution were expressed as means ± standard deviations between the two groups, and was compared with the independent-samples *t*-test. The chi-square test was used for gender data, P50 (P25, P75) was used to measure the PPPM score of pharyngeal pain on the third, fourth, fifth, sixth and seventh days after surgery, the degree of pharyngeal swelling on the fourth, fifth, sixth and seventh days after surgery, and the change of body weight. Mann–whitney *U* test was used to compare the pain scores at each time point between the two groups. Kruskal–wallis test was used to compare pain scores at each time point between multiple groups, and *p* < 0.05 was considered statistically significant.

## Results

### Pain

The PPPM scores from Day 0 to Day 7 were lower in the tonsillotomy group than in the tonsillectomy group (Table 2).

**Table 2:** Pharyngeal pain (PPPM scores) comparisons between the tonsillotomy and tonsillectomy groups.

Day	Tonsillotomy group (*n* = 63)	Tonsillectomy group (*n* = 58)	t/z	*P*
D0	4.29 ± 1.38	7.60 ± 1.38	−13.15 (t)	0.000
D1	3.40 ± 1.77	7.19 ± 1.61	−12.259 (t)	0.000
D2	1.63 ± 1.22	5.72 ± 1.89	−14.19 (t)	0.000
D3	2 (0,2)	4 (3,5.25)	−7.610 (z)	0.000
D4	2 (0,2)	4 (2,6)	−7.154 (z)	0.000
D5	1 (0,2)	3 (2,3)	−7.076 (z)	0.000
D6	1 (0,2)	2 (2,4)	−4.075 (z)	0.000
D7	0 (0,1)	2 (2,4)	−4.138 (z)	0.000

### Degree of pharyngeal swelling

In both groups, swelling of the pharyngeal cavity was greatest on Days 0 and 1 and gradually decreased from Day 2 to Day 7. The tonsillotomy group exhibited significantly less swelling than the tonsillectomy group (Table 3).

**Table 3:** Comparisons of pharyngeal swelling between the tonsillotomy and tonsillectomy groups.

Day	Tonsillotomy group (*n* = 63)	Tonsillectomy group (*n* = 58)	t/z	*P*
D0	2.14 ± 0.64	3.66 ± 0.66	−12.717 (t)	0.000
D1	1.70 ± 0.89	3.52 ± 0.77	−11.914 (t)	0.000
D2	0.87 ± 0.63	2.78 ± 0.89	−13.531 (t)	0.000
D3	0.63 ± 0.57	1.93 ± 0.95	−9.136 (t)	0.000
D4	1 (0,1)	2 (1,3)	−6.856 (z)	0.000
D5	1 (0,1)	1 (1,2)	−5.421 (z)	0.000
D6	0 (0,1)	1 (1,2)	−5.583 (z)	0.000
D7	0 (0,1)	1 (1,2)	−5.290 (z)	0.000

### Weight

Compared with the preoperative weights, both groups had lost weight on the 7th postoperative day. Body weight decreased by 1 (0, 1.5) kg in the tonsillotomy group and by 2 (1.43, 2.5) kg in the tonsillectomy group. The difference in weight change between the two groups was statistically significant.

### Pain in preschool and school-age children

**Figure 1:**
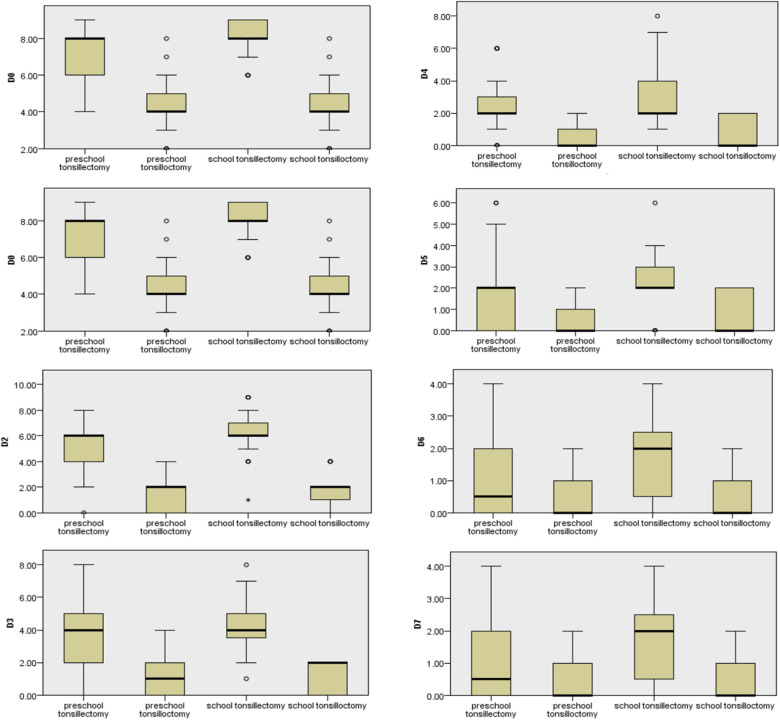
The comparison of pharyngeal pain (PPPM scores) among preschool-age tonsillotomy, preschool-age tonsilletomy, school-age tonsillotomy, and school-age tonsilletomy from D0∼D7.

The two groups of patients were divided into 4 groups according to age: preschool tonsillotomy group, preschool tonsillectomy group, school-age tonsillotomy group, and school-age tonsillectomy group. On the day of surgery and on the first, second, third, and fourth postoperative days, there were statistically significant differences in the PPPM scores between the preschool tonsillotomy and preschool tonsillectomy groups (*p* < 0.05). There were also statistically significant differences between the PPPM scores of the school-age tonsillotomy and school-age tonsillectomy groups on the same days (*p* < 0.05). On postoperative days five, six, and seven, there were no statistically significant differences between the PPPM scores of the preschool tonsillotomy group and those of the preschool tonsillectomy group (*p* > 0.05); however, there were statistically significant differences between the PPPM scores of the school-age tonsillotomy group and those of the school-age tonsillectomy group (*p* < 0.05), as shown in Table 4 and Figure 1.

**Table 4:** Comparisons of pharyngeal pain (PPPM scores) in preschool and school-age children between the tonsillotomy and tonsillectomy groups.

Day	Preschool-age Tonsillotomy (*n* = 30)	Preschool-age tonsilletomy (*n* = 26)	School-age tonsillotomy (*n* = 33)	School-age tonsilletomy (*n* = 32)	*p*
D0	4 (4,5)	8 (6,8)	4 (4,5)	8 (8,9)	0.000
D1	4 (2,4)	8 (5.5,8)	4 (2,4)	8 (7,8)	0.000
D2	2 (0,2)	(3.75,6.25)	2 (1,2)	6 (6,7)	0.000
D3	1 (0,2)	4 (2,5.25)	2 (0,2)	4 (3.25,5.5)	0.000
D4	0 (0,1.25)	2 (2,3.25)	0 (0,2)	2 (2,4)	0.000
D5	0 (0,1)	2 (0,2)	0 (0,2)	2 (2,3.75)	0.000
D6	0 (0,1)	0 (0,2)	0 (0,1)	2 (1,2.75)	0.000
D7	0 (0,1)	0 (0,2)	0 (0,1)	2 (1,2.75)	0.000

## Discussion

OSA is a common disease among children. If not treated, it may cause serious complications, such as growth retardation, craniofacial deformity, and cognitive impairment (7). Adenoidectomy and/or tonsillectomy are the first-choice treatments (8, 9). Tonsillectomy is the traditional surgical method, but with the development of low-temperature plasma, lasers, and ultrasonic scalpels, tonsillotomy is increasingly used for the treatment of OSA in children. Several studies have shown that there is no significant difference in long-term efficacy between tonsilloctomy and tonsillectomy (6, 10–12).

In this study, we continuously observed the changes in pain and swelling of the pharyngeal cavity in the tonsillectomy and tonsillectomy groups over seven postoperative days and found that the PPPM scores in both groups were highest on the day of surgery and on the following day. Mild pain (PPPM < 6) from the day of tonsilloctomy surgery to the second day after surgery, The tonsillectomy group, patients exhibited severe pharyngeal pain (PPPM ≥6) on the first two days. Likewise, pharyngeal swelling was most severe on the first two days after the operation in both groups. The postoperative inflammatory response is related to swelling of the pharyngeal cavity caused by the release of inflammatory factors within 48 h (13). With the gradual reduction in the inflammatory response and edema, the pain begins to wane. The pain levels in the tonsillectomy group were mild (PPPM ≤4) from Day 3 to Day 7. The pain levels in the tonsillotomy group were significantly lower, and there was no obvious pain from Day 4 onward. This is because tonsillectomy was performed along the Capsule of tonsil, and the glossopharyngeal nerve and vagus nerve sensory fibers were densely present in the subcapsular muscle layer of the the tonsillar fossa, leading to stronger stimulation of the muscularis nerve. Navaneethan et al. (14) reported that the depth of thermal damage to tissue caused by a plasma radiofrequency knife was 0.7–0.8 mm. Tonsillotomy, on the other hand, retains part of the tonsil tissue, which acts as a surgical barrier, reducing neuromuscular stimulation and pain.

In this study, the patients The patients in the tonsillotomy group and the tonsilletomy group were divided into two subgroups of preschool age and school age according to age. On the 5th, 6th and 7th days after operation, compared with preschool children, partial tonsillectomy in school age children was more painful than total tonsillectomy. This may be because although patients aged 7–12 years have not yet developed chronic tonsillitis, as age increases, the frequency of tonsillitis may also increase. postoperative pains of school age children was obvious. Preoperative anxiety may also aggravate the postoperative pain of children undergoing surgery. Age is an important factor affecting preoperative anxiety in children, as it is related to their responses to the outside world (15). Preoperative parental comfort can effectively relieve preoperative anxiety in preschool children. On the other hand, informing school-age children undergoing total tonsillectomy about the degree and duration of postoperative pain may aggravate their preoperative anxiety and lead to severe postoperative pain.

Pain assessments in children have been based mostly on the visual analog scale. However, a previous study found that the PPPM correlated well with pain (16), and another study recommended its use for family assessments of pain after pediatric surgeries (17). In this study, we used the PPPM to observe postoperative behavioral changes in children. Sakki et al. (18) reported that both patients undergoing tonsillotomy and patients undergoing tonsillectomy lost weight postoperatively, with weight loss in the first week being greater among the latter. Our results are consistent with these findings. Weight loss may be related to the relevant dietary recommendations of the Clinical Practice Guidelines for Standardized Low-Temperature Plasma Radiofrequency Ablation Tonsillectomy and Adenoidectomy in Children (19) for partial or total tonsillectomy. About three weeks after the operation, the patient can return to a normal diet, depending on the wound's condition. Weight loss in the tonsillectomy group in this study was associated with insufficient food intake due to pain. In the tonsillotomy group, although the patients experienced mild postoperative pain, they also lost weight because their diet was restricted and changed.

In this study, compared to tonsillectomy, tonsillotomy was associated with less postoperative pain, which lasted for a shorter time, less weight loss, and faster recovery than tonsillectomy. Tonsillotomy reflects our enhanced recovery after surgery practice, which improves the treatment effects, reduces postoperative complications, and accelerates postoperative recovery, thereby shortening hospitalization times and reducing medical costs (20).

Compared with previous studies, ours is a prospective study, including 121 patients, that reduced the effect of bias. Previous studies have mostly used VAS or Wong–Baker FACES score, while we used PPPM score, which is related to pain. This approach resulted in a better correlation. In our study, we subgrouped patients according to age and analyzed the differences in pain patterns between the postoperative day 0 and postoperative day 7, the partial and total tonsillectomy groups, and the two age groups. Moreover, we observed swelling of the pharynx from the first to the seventh postoperative day in the partial and total tonsillectomy groups, which better explained the pain degree of the two groups of patients. This study has certain limitations. First, the sample size was small. Second, factors such as anxiety and differences in families' educational levels that may have affected the results were not considered.

In conclusion, the pain caused by tonsillotomy in children with OSA was obvious over the first 24 h after the operation and subsequently mild over the first postoperative week. The pain levels, pharyngeal swelling and weight loss associated with tonsillotomy were lower than those associated with tonsillectomy. On the fifth, sixth, and seventh postoperative days, compared with preschool children, tonsillotomy in school-age children is more advantageous in school-age children.

## Data Availability

The original contributions presented in the study are included in the article/Supplementary Material, further inquiries can be directed to the corresponding author/s.
